# Right ventricular apical versus septal pacing: impact on left ventricular synchrony and function

**DOI:** 10.1186/cc10792

**Published:** 2012-03-20

**Authors:** I Atteia, A Alazab, K Hussein, N Abeed, H Nagi

**Affiliations:** 1 Cairo University, Cairo, Egypt

## Introduction

Right ventricular apical pacing alters the LV activation resulting in an adverse effect on LV function and synchrony. On the contrary, RV septal pacing results in narrower QRS and may be more physiological with less deleterious long-term effect on LV echocardiographic and hemodynamic parameters.

## Methods

Forty patients indicated for permanent DDD pacing were studied. All patients were subjected to transthoracic echocardiography calculating LVESD, LVEDD, EF% and CO together with tissue Doppler imaging (TDI) to detect LV dyssynchrony. Patients were randomly classified into two groups, group I having RV apical pacing and group II having RV septal pacing. The acute threshold, R-wave sensing and fluoroscopic time were measured in all patients and compared in both groups. Both groups were followed-up over a period of 6 months.

## Results

QRS durations were significantly narrower in group II patients (148 ± 6.9 vs. 162 ± 6 ms, *P *= 0.001). Electrical parameters at the time of implantation were satisfactory for all patients (acute stimulation threshold was 0.5 ± 0.18 V; R wave sensing was 11 ± 1.6 mV and ventricular impedance was 630 ± 90 Ohm). No single patient needed ventricular lead repositioning. The acute pacing threshold, R-wave sensing, ventricular impedance and fluoroscopic time did not change significantly in both groups. During follow-up, it was found that in group II patients with RV septal pacing there was significantly lower LVESD (3.0 ± 0.6 vs. 3.4 ± 0.6 cm, *P *= 0.004), significantly higher LVEF% (69 ± 13 vs. 61 ± 8, *P *= 0.01), significantly higher CO (4.9 ± 0.3 vs. 4.5 ± 0.6 l), and significantly lower septal to lateral wall delay in LV using TDI (72 ± 5 vs. 83 ± 6 ms, *P *= 0.001) if compared to group I patients with RV apical pacing.

## Conclusion

Long-term RV septal pacing is feasible, and reliable with less adverse effects on LV synchrony and function when compared to RV apical pacing.
